# Establishing a large prospective clinical cohort in people with head and neck cancer as a biomedical resource: head and neck 5000

**DOI:** 10.1186/1471-2407-14-973

**Published:** 2014-12-17

**Authors:** Andrew Robert Ness, Andrea Waylen, Katrina Hurley, Mona Jeffreys, Chris Penfold, Miranda Pring, Sam Leary, Christine Allmark, Stu Toms, Susan Ring, Tim J Peters, Will Hollingworth, Helen Worthington, Chris Nutting, Sheila Fisher, Simon N Rogers, Steven J Thomas, The Head and Neck 5000 Study Team

**Affiliations:** National Institute for Health Research (NIHR) Biomedical Research Unit in Nutrition, Diet and Lifestyle at the University Hospitals Bristol NHS Foundation Trust and the University of Bristol and School of Oral and Dental Sciences, University of Bristol, Bristol, UK; School of Oral and Dental Sciences, University of Bristol, Bristol, UK; Surgical Research Team, University Hospitals Bristol NHS Foundation Trust, Bristol, UK; School of Social and Community Medicine, University of Bristol, Bristol, UK; National Cancer Research Institute Consumer Liaison Group (NCRI CLG) and Independent Cancer Patients Voice (ICPV), London, UK; MRC Integrative Epidemiology Unit and Avon Longitudinal Study of Parents and Children, School of Social and Community Medicine, University of Bristol, Bristol, UK; School of Clinical Sciences, University of Bristol, Bristol, UK; Cochrane Oral Health Group, School of Dentistry, University of Manchester, Manchester, UK; Royal Marsden Hospital and the Institute for Cancer Research, London, UK; Leeds Institute for Cancer and Pathology, University of Leeds, Leeds, UK; Evidence-Based Practice Research Centre (EPRC), Faculty of Health and Social Care, Edge Hill University, Ormskirk, Lancashire, UK

**Keywords:** Head and neck cancer, Clinical cohort, Prognosis research, Patient-reported outcomes, Sexual history, Quality of life, Biological samples

## Abstract

**Background:**

Head and neck cancer is an important cause of ill health. Survival appears to be improving but the reasons for this are unclear. They could include evolving aetiology, modifications in care, improvements in treatment or changes in lifestyle behaviour. Observational studies are required to explore survival trends and identify outcome predictors.

**Methods:**

We are identifying people with a new diagnosis of head and neck cancer. We obtain consent that includes agreement to collect longitudinal data, store samples and record linkage. Prior to treatment we give participants three questionnaires on health and lifestyle, quality of life and sexual history. We collect blood and saliva samples, complete a clinical data capture form and request a formalin fixed tissue sample. At four and twelve months we complete further data capture forms and send participants further quality of life questionnaires.

**Discussion:**

This large clinical cohort of people with head and neck cancer brings together clinical data, patient-reported outcomes and biological samples in a single co-ordinated resource for translational and prognostic research.

## Background

Head and neck cancer, though less common in developed countries, is an important cause of mortality and morbidity worldwide [1]. Survival is poor [2] and, despite advances in treatment, has not improved until recently [3]. The reasons for these recent improvements are unclear. They could include changes in disease aetiology or the fitness of people with disease, or alternatively, an improvement in treatment or alterations in lifestyle behaviour after treatment. The number of clinical trials carried out in people with head and neck cancer has increased over recent years but there is a need for observational studies to explore reasons for the improved survival and to identify predictors of outcome [4].

The importance of prognosis research has been highlighted recently [5, 6]. Some questions in prognostic research can be answered using routinely collected data or existing studies designed for other purposes [6]. Clinical cohorts, though expensive and time consuming, have a number of advantages over other study designs. These include the recruitment of a potentially broad and representative sample with limited exclusion criteria; participants with a shared (rather than staggered) clinical starting point; the measurement of prognostic factors not used in clinical practice; the inclusion of outcomes not routinely collected in existing sources (such as quality of life) and the collection of biological samples that can be analysed later. Many studies of disease prognosis have been small and their protocols have not been clearly described or reported [5]. There is therefore a need for well-designed, adequately-powered studies of this kind [5, 6].

In this paper we describe the methods for a large UK-based clinical cohort study in head and neck cancer called Head and Neck 5000. The primary justification for this study is to evaluate the impact of centralisation of care for people with head and neck cancer; a future publication will report on this evaluation. However, we also intend to develop a well-phenotyped clinical cohort that will provide a biomedical resource for translational and prognostic research in head and neck cancer.

## Methods

Head and Neck 5000 is an observational study that recruited people with head and neck cancer from across the United Kingdom. We describe the: process of designing and running the study; recruitment to the study; baseline data collection; blood and saliva samples; tissue samples; study follow-up; data management and statistical power. The study protocol, questionnaires, consent form and patient information leaflet were approved by the National Research Ethics Committee (South West Frenchay Ethics Committee, reference 10/H0107/57, 5^th^ November 2010) and subsequently approved by the research and development departments for all participating NHS Trusts. We will make copies of the study protocol and all documents described below available on the study website when recruitment finishes (http://www.headandneck5000.org.uk).

### Designing and running the study

We wanted to ensure that we drew on existing expertise and experience and at the same time encouraged collaboration and a sense of ownership of the study among clinicians treating people with head and neck cancer in the UK. To achieve this we had a number of preliminary discussions with key individuals and groups and hosted several workshops to bring together clinicians, methodologists and patient representatives. At these workshops we discussed and agreed both the principles and details of the study. Our aim was, as far as possible, to map the research protocol onto the treatment pathway of people with head and neck cancer and so minimise impact on both participants and clinicians. We also reduced the burden on participants by limiting the size and frequency of questionnaires, so as to encourage enrolment and continued participation. Before starting recruitment we completed a national survey of oncology centres and multi-disciplinary teams treating head and neck cancer that provided a picture of care nationally [7] We formally initiated centres before they opened to data collection and carry out visits to check on data quality. We have set up a study website, send regular newsletters to centres and provide them with individual reports on their recruitment and response rates.

### Recruitment to the study

All people with a new diagnosis of head and neck cancer are eligible to join the study. People with cancers in the pharynx, mouth, larynx, salivary glands and thyroid are all included. People with lymphoma, tumours of the skin or a recurrence of a previous head and neck cancer are excluded from the study. People have to be recruited before their treatment starts, unless their cancer treatment was is their diagnostic procedure. Where this is the case participants have to be recruited within a month of the diagnostic procedure. Potential participants for whom the decision is to provide palliative support are recruited as soon after diagnosis as possible. Potentially eligible people are identified by the multi-disciplinary team treating them. A a member of the clinical team in the local centre introduces the study to potential participants and either one of the clinical team or the research nurse give them a copy of the patient information leaflet. Participants are given an opportunity to consider this leaflet. They are then approached by a research nurse based in the local centre. The nurse answers any questions they have and then obtains written informed consent to participate in the study. This consent is wide-ranging and includes agreement to: collect, store and use biological samples; obtain samples of stored tissue; carry out genetic analyses and collect information from hospital notes and through record linkage. The teams keep logs of the number of eligible people not enrolled and the reason they were not recruited. The detailed process of recruitment is adapted as necessary by centres to ensure that it maps onto local practice in each centre.

### Baseline data collection

Having obtained informed consent the research nurse gives the participant three questionnaires to take away, complete and subsequently hand in to the clinic or return to the study centre in a pre-paid envelope. The research nurse offers to help complete any of the questionnaires where necessary. A more detailed summary of the contents of the questionnaires used at baseline and follow up is included in Table 1. The first questionnaire is five pages long and enquires about social and economic circumstances, overall health and lifestyle behaviours such as smoking and alcohol consumption. The second questionnaire is nine pages long and enquires about physical and psychological health, well-being and quality of life. The version of this questionnaire used in the Bristol centre contains an additional nine pages of questions on physical appearance. The third questionnaire comprises just one page and enquires about past sexual behaviours. The research nurse explains that this is because of the role of human papilloma virus infection in the aetiology of head and neck cancer to ensure that people understand its relevance and are not offended. Once the nurse has obtained consent s/he abstracts information on diagnosis, treatment and co-morbidity onto a short data capture form using questions based on a national audit [8]. Centres are encouraged to remind participants to complete questionnaires but this is not always possible (particularly where the time between consent and starting treatment is short). We code diagnosis using the International Classification of Diseases (ICD) version 10 [9]. We derive the clinical staging of the tumour from the T (characteristics of the tumour site), N (degree of lymph node involvement) and M (absence or presence of metastases) based on the American Head and Neck Society TNM staging of head and neck cancer [10].

**Table 1:** **Contents of questionnaires and data collection forms included in the head and neck 5000 clinical cohort study**

Questionnaire	Question set	Research topic	Number of pages	Questionnaire pack
Data capture form	Diagnosis and treatment	Based on the UK National Head and neck Cancer Audit [8]	1	Baseline, 4-month, 12-month
	Co-morbidity	Adult co-morbidity assessment 27 (ACE-27) [11]	1	
Health and Lifestyle	About You	Demographic data, Education, occupation [12], Income [13], EQ5D initially then EQ-5D-5 L [14], Smoking [15, 16], Alcohol [15, 16]	4	Baseline, 4-month, 12-month
Quality of life	Your Outlook	Revised Life Orientation Test (LOT-R) [17]	1	Baseline, 4-month, 12-month
	Your General Health	EORTC QLQ-C30 [18]	2.5	Baseline, 4-month, 12-month
	Specific Aspects of Your Health	EORTC QLQ-H&N35 [19]	2	Baseline, 4-month, 12-month
	Your Feelings	Hospital Anxiety and Depression Scale (HADS) [20]	2	Baseline, 4-month, 12-month
	Your Diet	Three items (fruit, vegetables and deep fried food) modified from the semi-quantitative Food, Frequency Questionnaire [21]	1	Baseline, 4-month, 12-month
	You and Cancer	Fears of Recurrence [22]	½	4-month, 12-month
	Your Personal costs	Designed by the study team	2	4-month , 12-month
Sexual history	Sexual History	Sexual History [23]	1	Baseline
Quality of life	Your symptoms	Head and neck radiotherapy questionnaire (late toxicity) [24]	5	12-months (only people who receive radiotherapy)
Withdrawal form	Withdrawal from study	Questions designed by the study team completed by research nurse	1	As appropriate
Mortality form	Place and mode of death	Questions designed by the study team completed by research nurse	2	As appropriate
Quality of life additional questions used in Bristol participants	Your Quality of Life	The revised University of Washington (UW) QOL questionnaire [25, 26]	2	Baseline, 4-month, 12-month
	Difficulties in Your Life	The Social Difficulties Inventory (SDI) [27]	2	Baseline, 4-month, 12-month
	Your Appearance	The Derriford Appearance Scale (DAS 24) [28]	6	Baseline, 4-month, 12-month

### Blood and saliva samples

Participants are asked to provide a blood sample and a saliva sample. The research nurse collects 16 ml of venous blood and puts this in two EDTA tubes (10 ml and 6 ml). For the saliva sample the local research nurse asks the participant to rinse their mouth and once saliva is flowing in their mouth to spit (at least 1 ml) into a sterile screw top container. The research nurse then posts the blood tubes and saliva container to the study centre laboratory at ambient temperature in pre-paid approved packaging, meeting UN Packaging Instruction PI650. The blood samples are spun at 3500 rpm for 10 minutes. The buffy coat layer is stored for future DNA extraction. Up to 8 ml of plasma in total is stored in a selection of 200 μl and 500 μl plasma aliquots. Saliva samples are divided into seven 1 ml samples. All samples are frozen and stored at −80°C in the Avon Longitudinal Study of Parents and Children (ALSPAC) bio-sample repository (http://www.bristol.ac.uk/alspac/). DNA extraction is being carried out by LGC genomics (http://www.lgcgenomics.com/). To date 2,000 buffy coat samples have been extracted using the Kleargene spin column extraction method (http://www.lgcgroup.com/products/dna-extraction-kits). Samples are eluted in 1 ml low salt buffer. DNA is quantified using picogreen, the mean DNA concentration is 97.21 ng/μl, (standard deviation 46 ng/μl), with a range of <10 ng/μl (5 samples) to 404 ng/μl. 1795 samples have a concentration >50 ng/μl.

### Tissue samples

We obtain tissue either from the diagnostic procedure or from the operation to remove the primary tumour. We follow a hierarchy of access protocol so that local research tissue banks have first access to tissue, with the study only receiving additional tissue where available. We ask the local pathologist to select one representative paraffin embedded tumour block from the primary site and if applicable, another from a matched lymph node metastasis. The local pathology department also send an anonymised copy of the participant’s histopathology report with the tissue blocks and provide some brief details on the sample.

### Study follow-up

We do not collect any further biological samples from participants. We send out follow-up questionnaire packs at four months and 12 months after the person joined the study. These questionnaires repeat many of the questions included at baseline apart from those enquiring about previous sexual behaviour. We have added questions on fear of recurrence at both four and 12 months and questions on late radio-toxicity at 12 months. The research nurses abstract updated information on diagnosis and treatment from the hospital medical record onto a short data capture form at four and 12 months. We flag study participants with the Health and Social Care Information Centre (HSCIC) and we receive regular notifications of subsequent cancer registrations and mortality among cohort members. Where someone has died we ask research nurses in study centres to complete a short questionnaire that enquires about the place and circumstances of death. When someone decides to withdraw from the study we ask the research nurses in study centres to complete a form giving the date, details and reason for withdrawal.

### Data management

The Bristol study team enters data from questionnaires and the data capture form onto a central database with automatic range and logic checks to reduce data entry errors. We identify missing or inconsistent data on the data capture forms, in particular where the initial diagnosis, stage or both are inaccurate or unclear. We check data for these fields against text descriptions and pathology reports to minimise errors and missing data. We contact study centres for further details where necessary. We are carrying out double data entry on a 10% random sample of questionnaires to establish the error rate and to identify key questionnaire sections that may require double data entry for the whole cohort.

### Power calculation

Our power calculation was based on survival differences across 4,000 participants. This allowed for exclusions of rarer cancer types, withdrawals from the study, incomplete data and loss to follow-up from the target total of 5,000 enrolled. We initially assumed that people would be recruited from 10 centres and allowed for clustering by centre in the power calculation. If 2 year mortality was 35% and two-sided alpha is 0.05 we calculated that we would have 80% power to detect a difference in survival of around five percentage points for an intra-class correlation coefficient (ICC) of 0.005 and of around seven percentage points for an ICC of 0.01 (according to an individual patient characteristic or a measure of the quality of care they received split at the median). We have updated our power calculation, based on our actual recruitment from 78 centres, which indicates that we will have 80% power to detect a difference in survival of around four percentage points for an ICC of 0.005 and of around five percentage points for an ICC of 0.01.

## Discussion

This large clinical cohort is successfully recruiting people with head and neck cancer from across the UK. It is on track to consent 5,000 people by the end of December 2014. The cohort recruits people before treatment starts and obtains wide-ranging consent, clinical information, self-reported socio-demographic, lifestyle and quality of life data and biological samples.

We invested considerable time building a national clinical consensus about the need for the study and in designing the protocol before we started our fieldwork. We opened to recruitment in a few centres initially to ensure the protocol ran smoothly. This meant that there was clinical support for the study and that the protocol was ready to be rolled out, but this did delay our start date and our initial rate of recruitment. The only specific problem we have encountered was when one clinician refused to allow people under his care to complete the questionnaire on sexual behaviour. We were aware that some people might find these questions sensitive so we had put them in a separate questionnaire that was handed out with a careful explanation from the research nurse.

We decided that all cancers treated by the head and neck multi-disciplinary team should be included in the study. We thought this would make the study easier to recruit to, as everyone was eligible. Even though we expect that numbers for some tumours will be modest, given the limited data on such tumours, we think that this will still be potentially valuable.

Participants are recruited by research staff employed by NHS trusts. The hospital trusts are reimbursed indirectly through the research networks. This means we have no direct control over staffing levels or performance but on the other hand we do not have to appoint, train or manage staff locally. As we have reported previously the process of recruiting to national clinical observational studies in the UK is not straightforward and is often delayed by local processes [29]. Trusts are reimbursed for the number of people they recruit and not on the response rate or the quality of the data they collect.

This study is a resource and we encourage future collaborations to ensure it is fully exploited. We are currently creating a detailed data dictionary and formalising access arrangements. Details of these, copies of study questionnaires and updates on recruitment will be made available on our study website (http://www.headandneck5000.org.uk/). Around half of UK centres (and most of the larger centres) treating people with head and neck cancer are contributing to this study.

This study has therefore also created a national framework with capacity to recruit people with head and neck cancer into clinical research.

In conclusion we are creating a large DNA-backed clinical cohort in people with head and neck cancer. As with any large scale study it has limitations in terms of recruitment rate and completeness of data. Nevertheless we believe it will make important contributions to the study of survival in people with head and neck cancer and to prognosis research more generally. We welcome collaboration and use of the resource.
